# The association between *CD28* gene rs3116496 polymorphism and breast cancer risk in Chinese women

**DOI:** 10.1042/BSR20170884

**Published:** 2017-12-12

**Authors:** Yuxiang Yan, Xianbo Zhang

**Affiliations:** Department of Surgical Oncology, Wenzhou Central Hospital,32 Dajianxiang Road, Wenzhou, Zhejiang, China

**Keywords:** breast cancer, bioinformatics analysis, CD28, polymorphism

## Abstract

T-lymphocyte activation plays an important role in suppressing the development of human cancers including breast cancer (BC). Cluster of differentiation 28 (CD28) is the primary T-cell costimulatory molecule and enhances T-cell activation and proliferation. To examine the role of CD28 gene polymorphism in BC, we conducted a case–control study involving 312 BC patients and 312 controls in a Chinese Han population. Bioinformatics analyses were conducted to analyze the expression level of CD28 and its association with overall survival (OS) of BC. Genotyping was performed using a custom-by-design 48-Plex single nucleotide polymorphism (SNP) Scan™ Kit. Our results indicated that CD28 mRNA level was down-regulated in the BC patients, whereas high expression of CD28 showed better OS for BC. In addition, an increased risk of BC was associated with the rs3116496 CC genotype of *CD28* gene (CC vs. TT). The significant association was also observed in the recessive model. In conclusion, CD28 may be a tumor suppressor gene and rs3116496 polymorphism of *CD28* gene showed positively correlation with the increased risk of BC. However, larger studies with more diverse ethnic populations are needed to confirm these results.

## Introduction

Breast cancer (BC) is the most frequently diagnosed cancer among women and it is the leading cause of female cancer-related death [1]. In 2017, 255,180 new BC cases and 41,070 BC deaths are estimated to occur in United States [2]. Although the mechanism of BC is poorly understood, it has been widely accepted that environmental factors, genetic factors, and their interactions could contribute to the risk of BC [3]. It has been demonstrated that breast cancer associated gene 1 (BRCA1) gene mutations were associated with the high incidence of BC [4].

BC cells could escape immune surveillance by suppressing the T-cell response [5]. T-cell activation is dependent on the balance of costimulation and coinhibition [6]. Cluster of differentiation 28 (CD28), constitutively expressed on the majority of T cells, is the primary T-cell costimulatory molecule and enhances T-cell activation and proliferation [7]. In addition, B7 family members were identified as the coinhibitory molecular, such as cytotoxic T-lymphocyte antigen 4 (CTLA-4) and programmed cell death 1 ligand 1 (PD-L1) [8,9]. Isitmangil et al. [10] found that the levels of CD28 were significantly higher in BC patients than in the control group. Coinhibitory molecular CTLA-4 blockade has been demonstrated to enhance antitumor immunity [11]. CD28 is encoded at the same chromosomal loci as CTLA4 and thus has 31% amino acid homology, and interacts closely with CTLA4 [12]. Based on these observations, CD28 was associated with the development of BC by affecting the T-cell function.

Recently, two studies investigated the association between *CD28* gene polymorphism and BC risk [10,13]. Chen et al. [13] found that *CD28* rs3116496 polymorphism increased the risk of BC in a Chinese population. Isitmangil et al. [10] conducted another case–control study in a Turkish population and failed to detect significant association between rs3116496 polymorphism and BC risk. Different genetic backgrounds may contribute to the divergence. Therefore, we conducted a hospital-based case–control study with 312 BC patients and 312 controls to validate the conclusion in a Chinese Han population of Zhejiang province.

## Patients and methods

### Study subjects

We obtained approval of the study protocol from the Ethics Committee of Wenzhou Central Hospital (Zhejiang, China). All subjects provided written informed consent before participating in the study.

BC patients (312) were recruited from the Wenzhou Central Hospital (Zhejiang, China) between September, 2013 and January, 2016. Individuals with previous cancer history and metastasized cancer from other organs were not included in the present study. Cancer-free controls, frequency-matched to the cases on age were randomly selected from the same institutions during the same time period. Each patient was interviewed by trained personnel using a pretested questionnaire to obtain information on demographic data and related risk factors for BC. After the interview, 5 ml of peripheral blood was collected from each subject. The estrogen receptor (ER)/progesterone receptor (PR) information was obtained from the medical records of the hospitals.

### Oncomine analysis

Oncomine (www.oncomine.org), a cancer microarray database and web-based data mining platform, aims to stimulate new discovery from genome-wide expression analyses and compare the transcriptome data in most major types of cancer with respective normal tissues [14,15]. The filters were as follows: Gene: *CD28*; Analysis Type: Cancer vs. Normal Analysis; Cancer: Breast Cancer; Sample Type: Clinical Specimen. We got CD28 expression data in Breast from TCGA breast statistics.

### The Kaplan–Meier plotter

The prognostic significance of the mRNA expression of *CD28* gene in BC was evaluated using the Kaplan–Meier plotter (www.kmplot.com) [16]. With the purpose to assess prognostic value of a specific gene, the patient samples were divided into two cohorts according to the median expression of the gene (high vs. low expression). We analyzed the overall survival (OS) of BC patients by using a Kaplan–Meier survival plot. Log rank *P*-value and hazard ratio (HR) with 95% confidence intervals were calculated and displayed on the figure.

### Isolation of DNA and genotyping

Blood samples were collected using vacutainers and transferred to test tubes containing ethylenediaminetetraacetic acid (EDTA). Genomic DNA was isolated from whole blood using the QIAamp DNA Blood Mini Kit (Qiagen, Hilden, Germany). Genotyping was done by matrix-assisted laser desorption/ionization time-of-flight mass spectrometry (MALDI-TOF MS) using the Mass ARRAY system (Sequenom, San Diego, CA, U.S.A.).

### Statistical analyses

The demographic and clinical characteristics of study participants were evaluated by using the chi-squared test. Odds ratios (ORs) and 95% confidence intervals (CIs) were used to estimate the association between *CD28* gene rs3116496 polymorphism and risk of BC by logistic regression analyses. Hardy–Weinberg equilibrium (HWE) for *CD28* genotype distributions in controls was tested by a goodness-of-fit chi-squared test. All statistical analyses were performed using the SAS software package (var. 9.1.3; SAS Institute, Cary, NC, U.S.A.). *P*<0.05 was considered to indicate a significant difference.

## Results

### Oncomine analysis and prognosis analysis

**Figure 1:**
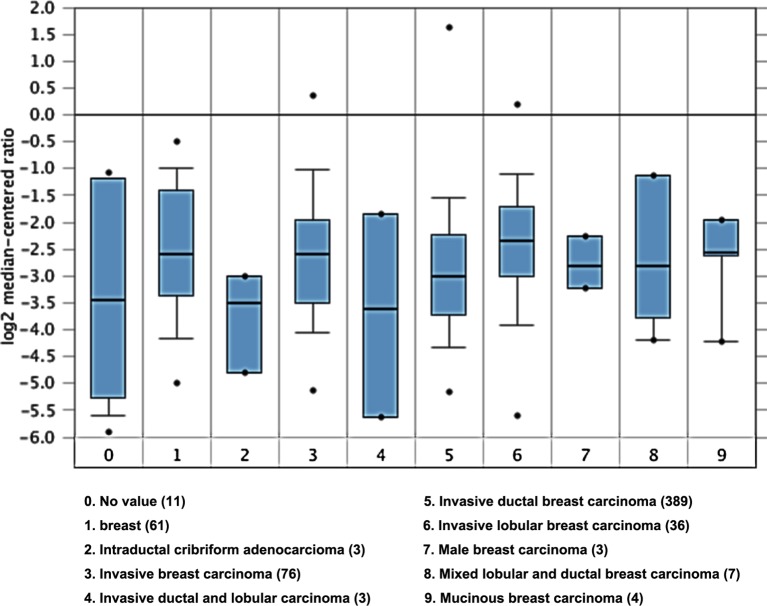
CD28 gene expression is down-regulated in BC compared with that in normal breast tissues The Student’s *t*-test was conducted using the Oncomine software (www.oncomine.org). The boxes represent the 25th through 75th percentiles. The horizontal lines represent the medians.

Using Oncomine analysis, we investigated mRNA levels of *CD28* gene in BC. As is shown in Figure 1, 0 for no value, 1 for the control group, and 2–9 for different types of BC. Three types of BC with larger sample size could present more reliable data. They were invasive BC, invasive ductal breast cancer (IDC), and invasive lobular breast cancer (ILC). The number of down-regulation datasets was found more than the number of up-regulation datasets. Overall, *CD28* gene was down-regulated in BC compared with normal breast tissue.

**Figure 2:**
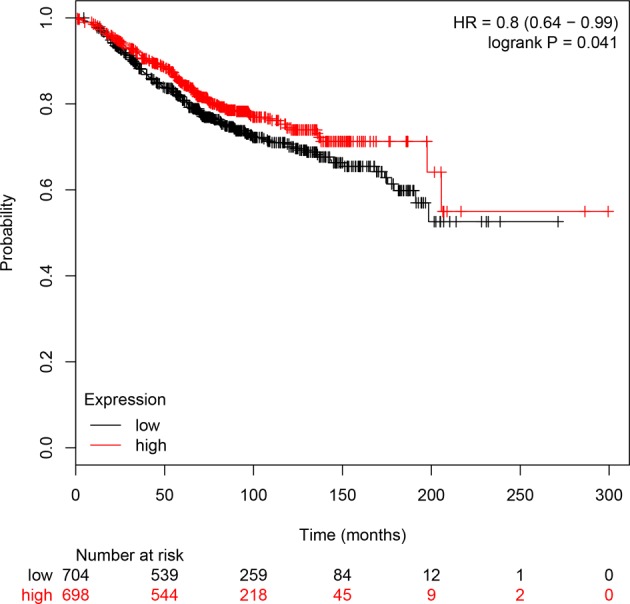
CD28 high expression is associated with better survival in BC Kaplan–Meier plots of overall survival: comparison of patients with high vs. low expression of CD28 in BC patients. The Kaplan–Meier plots were generated by Kaplan–Meier Plotter (http://www.kmplot.com).

Subsequently, we investigated the association between *CD28* gene expression and BC prognosis using a Kaplan–Meier plotter. Survival curves were plotted for all BC patients (Figure 2). CD28 high expression was found to be associated with better OS for all BC patients (HR, 0.8; 95%CI, 0.64–0.99; *P*=0.041). We speculated that *CD28* is a tumor suppressor gene, but more convincing studies are needed to validate the conclusions because these clinical data are from the database.

### Characteristics of the study population

The demographic and clinical characteristics of all subjects are listed in Table 1. A total of 312 BC patients and 312 healthy controls participated in the present study. Subjects were adequately matched for age and family history of cancer (*P*=0.882 and 0.650 respectively). The types of BC were mainly concentrated on invasive BC (7.7%), IDC (64.1%), and ILC (25.3%).

**Table 1:** Patient demographics and risk factors in breast cancer

Variable	Cases (*n*=312)	Controls (*n*=312)	*P*
Age (years)	51.71 ± 14.30	51.88 ± 13.84	0.882
Family history of cancer			
No	232(74.4%)	227(72.8%)	0.650
Yes	80(25.6%)	85(27.2%)	
Histology			
Invasive ductal breast cancer	200(64.1%)	–	
Invasive lobular breast cancer	79(25.3%)	–	
Invasive breast cancer	24(7.7%)	–	
Others	9(2.9%)	–	
Estrogen receptor			
Positive	193(61.9%)	–	
Negative	119(38.1%)	–	
Progesterone receptor			
Positive	204(65.4%)	–	
Negative	108(34.6%)	–	

### Quantitative analysis

The genotypes of *CD28* rs3116496 in two groups were summarized in Table 2. The genotype distribution in the controls conformed to HWE (*P*=0.083), suggesting these subjects could represent the total population. Carriers with genotype CC were at 2.15 times higher risk of BC than those with genotype TT (OR = 2.15, 95% CI = 1.02-4.51, *P*=0.044). Moreover, *CD28* rs3116496 polymorphism achieved significant difference in the association with BC in the recessive model (*P*=0.040), but not in the dominant model (*P*=0.504). The C allele of rs3116496 polymorphism exhibited no significant association with the risk of BC.

**Table 2:** Logistic regression analysis of associations between CD28 rs3116496 polymorphism and risk of breast cancer

Genotype	Cases* (*n*=312)		Controls* (*n*=312)		OR (95% CI)	*P*
	*n*	%	*n*	%		
TC vs. TT	67/217	21.5/69.5	71/223	22.8/71.5	0.97 (0.66–1.42)	0.875
CC vs. TT	23/217	7.4/69.5	11/223	3.5/71.5	**2.15 (1.02–4.51)**	0.044
CC vs. TC vs. TT						
TC + CC vs. TT	90/217	28.8/69.5	88/223	28.2/71.5	1.13 (0.79–1.61)	0.504
CC vs. TC + TT	23/284	7.4/91.0	11/294	3.5/94.2	**2.16 (1.04–4.52)**	0.040
C vs. T	113/501	18.1/80.3	99/517	15.9/82.9	1.19(0.88–1.58)	0.279

Bold values are statistically significant (*P*<0.05).

* The genotyping was successful in 307 cases and 305 controls.

## Discussion

In the present study, we found that *CD28* gene expression is down-regulated in BC patients, whereas CD28 high expression is associated with better OS for BC by bioinformatics analysis. In addition, CC genotype of rs3116496 polymorphism showed positively correlation with the increased risk of BC.

Tumor-specific T-cell response was beneficial to limiting the development of cancer, which was influenced by costimulatory and coinhibitory signals [17]. As one of the best characterized costimulatory molecules, CD28 competes with CLTA-4 (coinhibitory molecules) for B7 binding to enhance T-cell proliferation [18]. Therefore, *CD28* gene mutations may break the balance between costimulatory and coinhibitory molecules and changed the susceptibility of cancer.

Recently, the association between *CD28* rs3116496 polymorphism and cancer risk has been widely investigated, such as cervical cancer [19], non-small-cell lung cancer [20], colorectal cancer [21], BC [13], and renal cell carcinoma [22]. Only two studies evaluated the role of *CD28* rs3116496 polymorphism in BC risk [10,13]. In 2012, Chen et al. conducted a case–control study with 565 BC patients and 605 controls to investigate the association between *CD28* rs3116496 polymorphism and BC risk [13]. The results indicated that C allele of rs3116496 polymorphism increased the risk of BC in the northern Chinese population [13]. Isitmangil et al. [10] performed a case–control study in a Turkish population (79 cases and 76 controls) and failed to detect significant association between rs3116496 polymorphism and BC risk. In our study, CC genotype of rs3116496 polymorphism showed positive correlation with the increased risk of BC in the eastern Chinese population (312 cases and 312 controls). Obviously, the findings of studies from China (Asians) and Turkey (Caucasians) were different. We hypothesized that the following possible reasons may explain the different results of rs3116496 polymorphism between Asians and Caucasians. First, genetic heterogeneity for BC may exist in different populations. Second, the discrepancy may be explained by clinical heterogeneity between the different populations. Third, the sample size of the Caucasian population (79 cases and 76 controls) might not have been sufficiently large to reach a convincing conclusion when compared with Asian populations. Additionally, the different genotyping methods and random errors may also explain the different findings between Asians and Caucasians. It is noteworthy that we found CC genotype increased the risk of BC, while the study conducted by Chen et al. indicated C allele was associated with the risk of BC [13].

We attempted to make explanations for our positive results. First, the functional significance of rs3116496 polymorphism is unclear because it is located in the intron region. The significant association between *CD28* rs3116496 polymorphism and BC risk may reflect linkage disequilibrium with another potential functional variant or closely linked susceptibility gene. Second, the expression of CD28 was down-regulated in BC patients, showing worse OS for BC. Therefore, we guessed that *CD28* gene mutants may alter the expression of CD28, thus contributing the risk of BC by reducing T-cell response.

Several limitations in the present study need to be addressed. First, expression data and survival data of *CD28* are from the database, and we did not verify its validity by the present study. Second, the sample size of the present study is not large, which may get false-positive results. Third, other exposure information was missing, such as smoking and diet. Fourth, selection bias could not be avoided because the present study is a hospital-based case–control study. Fifth, we cannot add the experimental results *in vivo* and *in vitro* in the present study. Finally, we cannot make sure whether the location of rs3116496 polymorphism would affect the process of gene splicing.

In conclusion, the present study provides evidence that *CD28* gene was down-regulated in the BC patients, which was harmful to the survival of BC patients. In addition, we find that *CD28* rs3116496 polymorphism increases the risk of BC. However, our results were obtained from a moderate-sized sample, and larger, well-designed studies with ethnically diverse populations are warranted to further validate these findings.

## Abbreviations

BC: breast cancer
BRCA1: CD28
CD28: breast cancer associated gene 1
CTLA-4: cytotoxic T-lymphocyte antigen 4
ER: estrogen receptor
HR: hazard ratio
HWE: Hardy–Weinberg equilibrium
IDC: invasive ductal breast cancer
ILC: invasive lobular breast cancer
MALDI-TOF MS: matrix-assisted laser desorption/ionization time-of-flight mass spectrometry
OS: overall survival
PD-L1: programmed cell death 1 ligand 1
PR: progesterone receptor
SNP: single nucleotide polymorphism
